# Very low embryonic crude oil exposures cause lasting cardiac defects in salmon and herring

**DOI:** 10.1038/srep13499

**Published:** 2015-09-08

**Authors:** John P. Incardona, Mark G. Carls, Larry Holland, Tiffany L. Linbo, David H. Baldwin, Mark S. Myers, Karen A. Peck, Mark Tagal, Stanley D. Rice, Nathaniel L. Scholz

**Affiliations:** 1 Environmental and Fisheries Sciences Division, Northwest Fisheries Science Center, National Marine Fisheries Service, NOAA, 2725 Montlake Blvd. E., Seattle, WA 98112; 2 Habitat Assessment and Marine Chemistry Program, Auke Bay Laboratories, Alaska Fisheries Science Center, National Marine Fisheries Service, National Oceanic and Atmospheric Administration, 17109 Pt. Lena Loop Rd., Juneau, AK 99801.

## Abstract

The 1989 Exxon Valdez disaster exposed embryos of pink salmon and Pacific herring to crude oil in shoreline spawning habitats throughout Prince William Sound, Alaska. The herring fishery collapsed four years later. The role of the spill, if any, in this decline remains one of the most controversial unanswered questions in modern natural resource injury assessment. Crude oil disrupts excitation-contraction coupling in fish heart muscle cells, and we show here that salmon and herring exposed as embryos to trace levels of crude oil grow into juveniles with abnormal hearts and reduced cardiorespiratory function, the latter a key determinant of individual survival and population recruitment. Oil exposure during cardiogenesis led to specific defects in the outflow tract and compact myocardium, and a hypertrophic response in spongy myocardium, evident in juveniles 7 to 9 months after exposure. The thresholds for developmental cardiotoxicity were remarkably low, suggesting the scale of the Exxon Valdez impact in shoreline spawning habitats was much greater than previously appreciated. Moreover, an irreversible loss of cardiac fitness and consequent increases in delayed mortality in oil-exposed cohorts may have been important contributors to the delayed decline of pink salmon and herring stocks in Prince William Sound.

The year 2014 marked the 25^th^ anniversary of the 1989 Exxon Valdez oil spill in Prince William Sound, Alaska. At the time, the spill was the largest in U.S. history, with extensive oiling of shoreline spawning habitats for Pacific herring (*Clupea pallasi*) and pink salmon (*Oncorhynchus gorbuscha*), the two most important commercial fish species in the Sound. Herring larvae sampled in proximity to oil were visibly abnormal^1^, and mortality rates were higher for pink salmon embryos at oiled sites^2^. The herring fishery collapsed 3–4 years after the spill^3^,^4^ when the cohort spawned in oiled areas would have reached reproductive maturity^5^. The contribution of the spill to the herring population collapse, if any, was never determined and remains controversial.

In the early 1990s little was known about the effects of low-level crude oil exposures on fish early life stages. In the ensuing years, the syndrome of developmental defects and mortality documented in field-collected herring and salmon larvae was linked to polycyclic aromatic hydrocarbons (PAHs), an abundant fraction of most crude oils^6^,^7^,^8^. Subsequent research using the zebrafish model showed that the etiology of the syndrome was a disruption of embryonic cardiac function and morphogenesis^9^,^10^. This cardiotoxicity was specifically attributed to three-ringed PAHs^9^,^10^, and it was further shown that normal-appearing zebrafish embryos surviving trace crude oil exposures grow into adults with malformed hearts and reduced cardiorespiratory performance^11^. It is now established that crude oils from different geological sources disrupt heart development^12^,^13^ in a diversity of fish species^14^,^15^, by a mechanism involving the blockade of potassium and calcium ion channels essential for excitation-contraction (E-C) coupling in heart muscle cells^16^.

The crash of the Prince William Sound herring fishery suggests that shore-spawning fish species may have experienced a form of delayed mortality that went undetected in early toxicity assessment studies. Consistent with this, recruitment for the pink salmon population subsegments that spawned in oiled habitats in 1989 was poor^2^. Also, mark and recapture experiments found significant reductions (~40%) in juvenile to adult survival among pink salmon exposed transiently as embryos, despite having an outwardly normal appearance when released to the ocean^17^,^18^. The complex anatomical formation of the heart depends critically on normal pumping action during embryogenesis^19^,^20^, and respiratory-dependent behaviors (e.g., swimming) are critical determinants of early survival in marine fish^21^. Therefore, developmental exposures to oil-derived tricyclic PAHs may have impaired the cardiorespiratory fitness of herring and pink salmon at later life stages. To explore this, we transiently exposed herring and pink salmon embryos to trace concentrations of Alaska North Slope crude oil (ANSCO) and examined cardiorespiratory function, cardiac anatomy and histology in juveniles after 7–8 months of growth in clean seawater.

## Results

### Embryonic accumulation of PAHs

**Figure 1:**
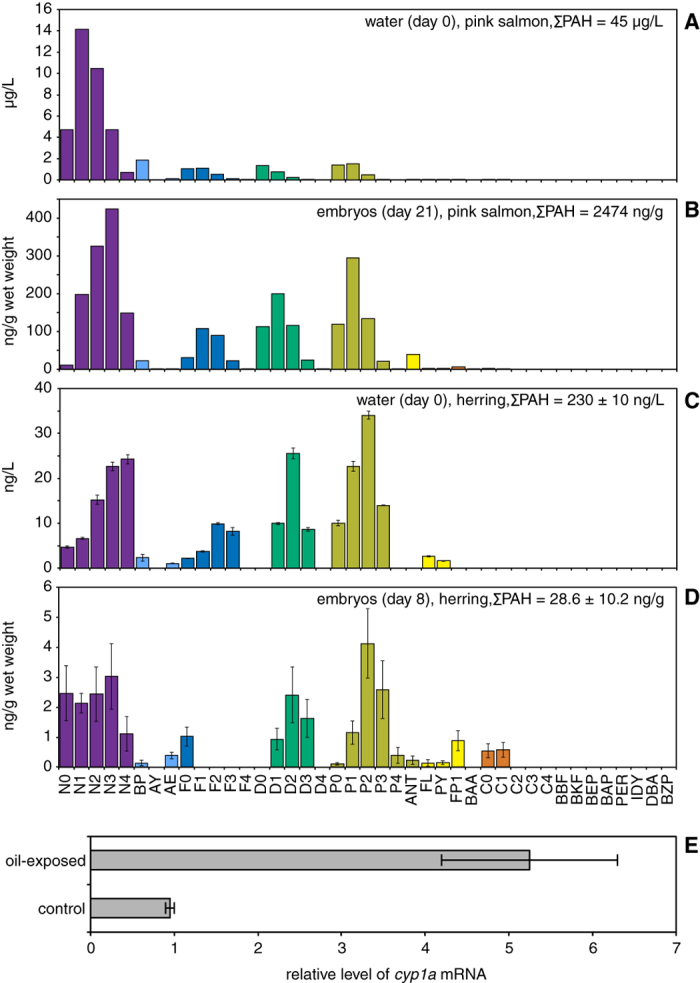
Uptake of PAHs into embryos and induction of *cyp1a*. Individual PAHs measured in water (**A**,**C**) and embryos (**B**,**D**). (**A**) Highest exposure concentration for pink salmon embryos (single water sample). (**B**) Pink salmon embryos at exposure day 21 (single pooled sample, 7 g wet weight). (**C**) Single exposure concentration for Pacific herring embryo exposure (triplicate water samples, mean ± s.e.m.). (**D**) Herring embryos at exposure day 8 (mean ± s.e.m.; *N* = 3 pooled samples, 1–3 g wet weight each). (**E**) Levels of *cyp1a* mRNA in herring embryos exposed to gravel effluents measured by QPCR as described under Methods. Data are mean ± s.e.m. of *cyp1a* levels normalized to *ef1α* levels measured in two replicates from each treatment with ~150 embryos each. N, naphthalenes; BP, biphenyl; AY, acenaphthylene; AE, acenaphthene; F, fluorenes; D, dibenzothiophenes; P, phenanthrenes; ANT, anthracene; FL, fluoranthene; PY, pyrene; FP, fluoranthenes/pyrenes; BAA, benz[*a*]anthracene; C, chrysenes; BBF, benzo[*b*]fluoranthene; BKF, benzo[*j*]fluoranthene/benzo[*k*]fluoranthene; BEP, benzo[e]pyrene; BAP, benzo[a]pyrene; PER, perylene; IDY, indeno[1,2,3-*cd*]pyrene; DBA, dibenz[*a*,*h*]anthracene/dibenz[*a*,*c*]anthracene; BZP, benzo[*ghi*]perylene. Parent compound is indicated by a 0 (e.g., N0), while numbers of additional carbons (e.g. methyl groups) for alkylated homologs are indicated as N1, N2, etc.

Salmon and herring embryos were exposed to water-soluble components of ANSCO using oil-coated gravel generator columns, beginning shortly after fertilization and ending within the organogenesis phase after completion of key steps of early heart development (~60% of embryogenesis, Supplementary Fig. S1). For salmon, initial aqueous ΣPAH concentrations were linearly related to nominal oil load (Supplementary Fig. S2A), resulting in an exposure range of 0.2  μg/L (control) through 45.4  μg/L (Table 1), with an exponential drop in ΣPAH concentrations for all oil treatments during exposure (Supplementary Fig. S2B). For herring, column flow was initiated three weeks before embryo exposure started, and initial aqueous ΣPAH concentrations were 0.230 ± 0.010  μg/L and 0.039 ± 0.003  μg/L for oiled and clean gravel, respectively (Table 1). More prolonged weathering (relative to the salmon exposure) was evident as a greater depletion of parent and C1-naphthalenes, fluorenes, dibenzothiophenes, and phenanthrenes, with higher proportions of C2-alkyl homologs (Fig. 1C). In general embryos of both species accumulated patterns of individual PAHs that mirrored the PAH composition of water (Fig. 1 and Supplementary Fig. S3). Pink salmon embryos accumulated PAHs in a concentration-dependent manner (Supplementary Fig. S3), with tissue ΣPAH levels ranging from 26 ng/g wet weight (control) to 2474 ng/g (Table 1), while herring embryos accumulated ΣPAH concentrations of 28.6 ± 10.2 ng/g wet weight and 9.3 ± 3.7 ng/g wet weight from oiled and control gravel effluent, respectively (Table 1, Fig. 1D). This very low level PAH exposure in herring was nevertheless sufficient to cause a 5-fold induction of *cyp1a* mRNA, encoding the PAH-metabolizing enzyme cytochrome P4501A (Fig. 1E).

Although PAH accumulation was much higher in salmon relative to herring on the basis of wet weight (Table 1), salmon embryos are much larger with higher lipid content. Lipid correction more closely aligned tissue ΣPAH concentrations between herring (1787 ± 256 ng/g lipid) and the lowest salmon dose (2066 ng/g lipid) relative to their respective controls (582 ± 178 and 240 ng/g lipid; Table 1). Therefore, despite an approximately 40-fold difference in aqueous exposure concentrations, the two treatments produced similar tissue concentrations, and hence could be expected to produce overlapping biological effects.

### Low levels of visibly malformed embryos

Morphological defects were assessed in hatched pink salmon alevins and dechorionated herring embryos on the day of transfer to clean water (Supplementary Table S1). Only low levels of overt cardiotoxicity were evident as mild pericardial edema in 11% of pink salmon embryos (high dose) and 11.8 ± 3.2% of oil-exposed herring embryos. For pink salmon, minor edema at the posterior end of the yolk sac was more frequent than pericardial edema, albeit not dose-dependent (Supplementary Table S1). Minor hemorrhages in the head or trunk were also observed in a small percentage of oil-exposed alevins. Herring embryos were evaluated at a stage prior to blood cell formation, and thus corresponding effects of oil on vascular integrity were not assessed. Overall, the targeted exposure concentrations yielded minimal acutely lethal toxicity (e.g., overt heart failure), as intended.

### Reduced juvenile growth

Oil exposure reduced the growth rate for juvenile pink salmon. At emergence (the end of yolk absorption), juvenile mass among treatments was approximately the same (0.2 g), and the size of controls and fish from the highest dose were indistinguishable (P_ANOVA_ = 0.914). Subsequent growth was exponential in each treatment but slower with increasing dose. Specific growth rates (Supplementary Fig. S4) as measured by both mass and length, declined significantly with dose (mass r = −0.954, P_linear regression _= 0.012; length r = −0.912, P_linear regression _= 0.031).

### Reduced juvenile cardiorespiratory function

**Figure 2:**
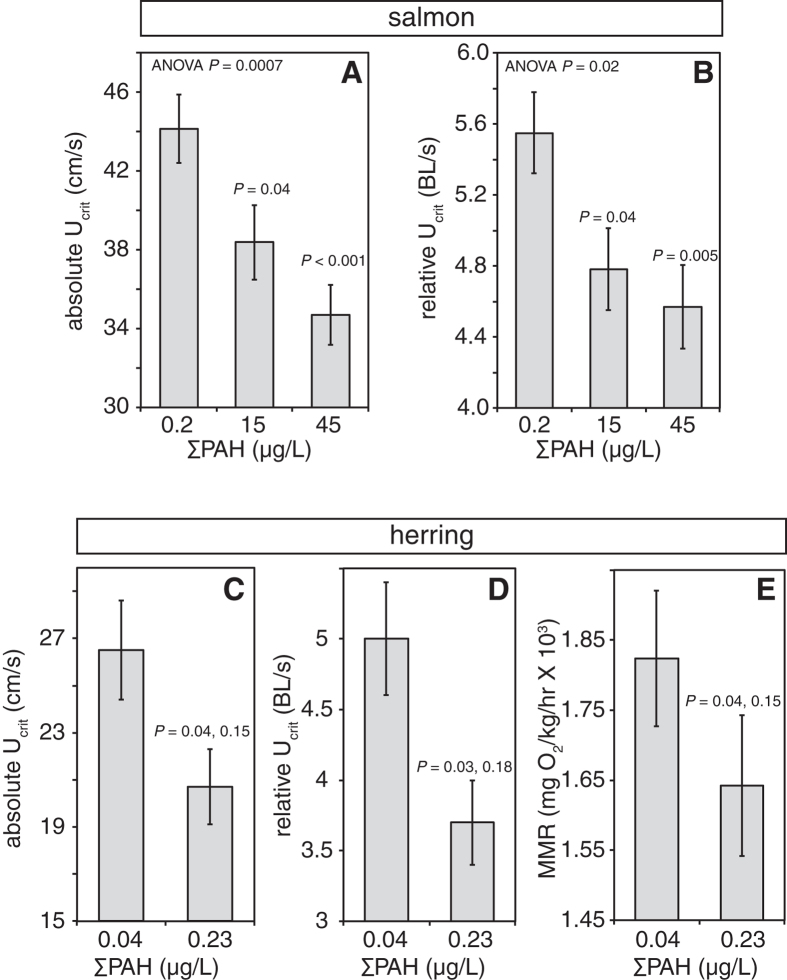
Embryonic oil exposure reduced critical swimming speed and maximum metabolic rate of juveniles. Mean *U*_crit_ (±s.e.m.) is given as an absolute speed (**A** and **C**, cm/s) or relative to body length (**B** and **D**, BL/s) for (**A**,**B**) salmon exposed to clean effluent (ΣPAH 0.2  μg/L, *N* = 51) or oiled gravel effluent with ΣPAH 15  μg/L (*N* = 45) and 45  μg/L (*N* = 52); and (**C**,**D**) herring exposed to clean gravel effluent (ΣPAH 0.04  μg/L, *N* = 32) and oiled gravel effluent with ΣPAH 0.23  μg/L (*N* = 33). (**E**) Maximum metabolic rate (MMR) for juvenile herring during *U*_crit_ assays. Oxygen consumption data passing statistical criteria (clean gravel, *N* = 23; oiled gravel, *N* = 22) were used to calculate MMR as described under Methods. For salmon data, *P* values are shown for effect of oil exposure from ANOVA while *P* values over oil-exposed groups represent comparison to controls in post-hoc analysis. For herring data, *P* values shown are for effect of treatment (oil exposure) and tank effect, respectively, from a nested ANOVA (replicate nested under treatment).

We used critical swimming speed (*U*_*crit*_) as a measure of cardiorespiratory performance in juvenile pink salmon and Pacific herring. *U*_*crit*_ is the maximum sustained swimming speed reached at the point of fatigue and at which maximum sustained oxygen uptake occurs^22^. A swim tunnel respirometer designed for small fish (4–12 g) was used to measure *U*_*crit*_ for both species at 8–9° C following seven to eight months of growth in clean water. For both species, *U*_*crit*_ was significantly reduced in juveniles exposed to oil during embryonic development (*P* < 0.001; Fig. 2). For pink salmon, *U*_*crit*_ was determined for three treatments (control, ΣPAH 15.4  μg/L and 45.4  μg/L exposures). Absolute *U*_*crit*_ was greatest in control fish (44.1 ± 1.7 cm/sec) and lowest in fish from the ΣPAH 45.4  μg/L exposure (34.7 ± 1.5 cm/sec, Fig. 2A). Although there was a weakly positive correlation between fish length and *U*_*crit*_ in juvenile pink salmon (r = 0.394; Fig. S5A), the slope of the linear regression was significant (*P* < 0.001). Fish from the high oil treatment were significantly shorter (*P* = 0.002) and lighter (*P* = 0.006) relative to controls (Supplementary Fig. S5B, C). Fish also grew during the month-long data collection window (Supplementary Fig. S5D; *P*_ANCOVA_ < 0.001). Although the slopes for the two oil treatments were equal (*P*_ANCOVA_ = 0.915), the controls were bigger on a given date (*P*_ANCOVA_ = 0.001). However, dose-dependent reduction in swimming speed was not entirely explained by differences in fish size, in particular because the smaller oil-exposed fish had lower relative *U*_*crit*_. Critical swimming speed normalized by length (relative *U*_*crit*_) produced the same dose-dependent pattern and significance (Fig. 2B), and relative *U*_*crit*_ in the ΣPAH 45.4  μg/L treatment group (4.6 ± 0.2 BL/sec) was significantly less than in controls (5.5 ± 0.2 BL/sec, *P* = 0.02). Reduction in relative *U*_*crit*_ was also significant after accounting for time and growth using 2-factor ANOVA models that included either length and *U*_*crit*_ or time and *U*_*crit*_.

Absolute *U*_*crit*_ in juvenile herring was 26.5 ± 2.1 cm/sec for controls and 20.7 ± 1.6 cm/sec for oil-exposed (*P* = 0.04, ANOVA with tank nested in treatment; Fig. 2C). Oil-exposed herring were larger than controls (length 5.6 ± 0.1 cm vs. 5.3 ± 0.1, respectively; weight 1.6 ± 0.1 g vs. 1.3 ± 0.1 g). This was possibly due to enhanced juvenile growth at lower densities (higher in-tank mortality in the oil-exposed groups), further suggesting that smaller size of oil-exposed pink salmon was not responsible for reduced absolute *U*_*crit*_. The relative *U*_*crit*_ of exposed juvenile herring was significantly reduced from 5.0 ± 0.4 BL/sec (controls) to 3.7 ± 0.3 BL/sec (*P* = 0.03, ANOVA with tank nested in treatment; Fig. 2D). There was no tank effect for either measure (*P* = 0.15 and 0.18, respectively). Oxygen consumption was measured during herring swim trials and used to calculate maximum metabolic rates (Fig. 2E). Oil exposure significantly reduced maximum metabolic rate as fish approached *U*_*crit*_, from 1824 ± 96 mg O_2_/kg/hr in controls to 1642 ± 100 mg O_2_/kg/hr for oil-exposed (*P* = 0.04; tank effect *P* = 0.15), but did not significantly affect overall swimming efficiency as measured by cost of transport (controls 1.36 ± 0.15 J/kg/BL, oil-exposed 1.67 ± 0.15 J/kg/BL; *P* = 0.2). The standard metabolic rate was not determined, preventing a quantification of aerobic scope. However, these findings are consistent with reduced aerobic capacity and cardiac output during sustained swimming^23^.

### Altered cardiac structure in juveniles

**Figure 3:**
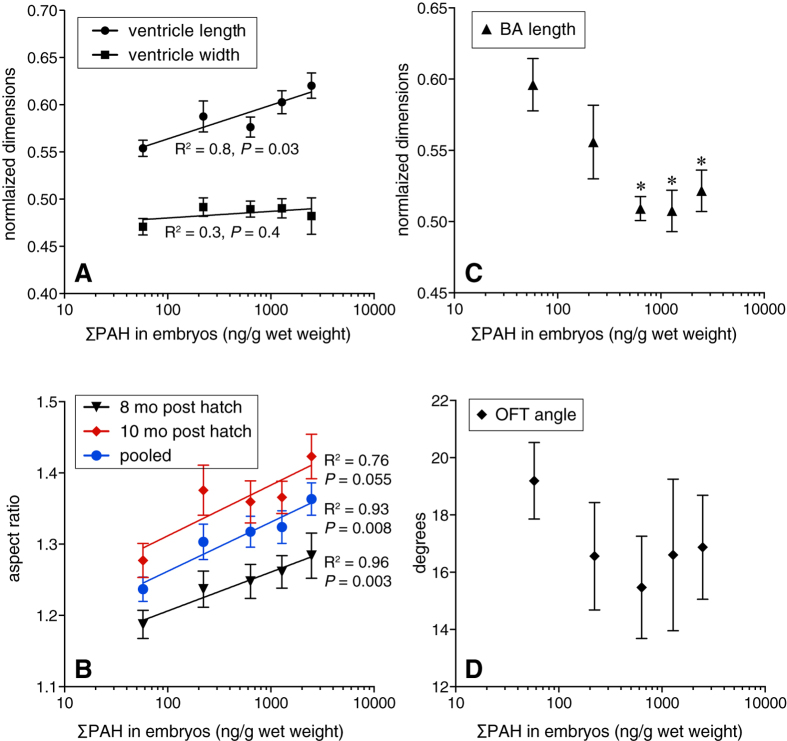
Dose-dependent changes in juvenile salmon cardiac morphology following embryonic oil exposure. Anatomical locations of measurements are shown in Supplementary Fig. S7. (**A**) Ventricular length and width normalized to fish fork length measured in juveniles 8 months after exposure (mean ± s.e.m.). Data were fit to a linear regression model; *P* value indicates significance of the slope. (**B**) Ventricular aspect ratio measured in juveniles 8 months (triangles) and 10 months (diamonds) after exposure, and both age groups pooled (circles) fit a linear regression model (mean ± s.e.m). *P* values indicate significance of slope. (**C**) Length of the bulbus arteriosus normalized to ventricular length in juveniles 8 months after exposure (mean ± s.e.m.). Data did not fit linear or non-linear regression models, but were highly significant by ANOVA (P = 0.0012); asterisks indicate doses significantly different from control (Dunnett’s test, α = 0.05). (**D**) Outflow tract angle in juveniles 8 months following exposure (mean ± s.e.m.). For 8 month fish, *N* = 12 for all oil exposed groups, 13 for control; for 10 month fish, *N* = 20–23 for all groups except lowest oil exposure dose, *N* = 11.

In addition to reducing swimming performance (and by proxy, aerobic capacity) in both species, embryonic oil exposure affected the eventual shape of the ventricle and outflow tract in juvenile hearts (Supplementary Fig. S6). Morphology of juvenile salmon ventricles was assessed after eight and ten months of growth in clean water. At eight months, there was a significant, ΣPAH dose-dependent increase in normalized ventricle length (R^2^ = 0.83, *P* = 0.03; Fig. 3A) but not width (R^2^ = 0.27, *P* = 0.4), resulting in a significant increase in ventricular aspect ratio (Fig. 3B; R^2^ = 0.96, *P* = 0.003). This persisted to ten months post-exposure (Fig. 3B), albeit with weaker significance (R^2^ = 0.76, *P* = 0.055). The length of the bulbus arteriosus (normalized to ventricular length) was reduced by oil exposure (Fig. 3C). Although not clearly dose-dependent, the effect of oil exposure was highly significant (ANOVA *P* = 0.0012) with salmon from all oil treatments having shorter outflow tracts relative to controls (all *P* values < 0.005) except for the lowest ΣPAH exposure (9.8  μg/L). While measurements of the outflow tract angle had greater variability, there was a clear trend that mirrored the effect on bulbus arteriosus length (Fig. 3D). Absolute dimensions of the ventricle were not significantly altered by oil exposure in a dose-dependent manner. Despite overall smaller body sizes in exposed fish, their ventricles tended to be larger than controls, with only the 30  μg/L exposure (12  μg/g tissue ΣPAH) group showing a significant increase in absolute length at 3.46 ± 0.08 mm relative to 2.96 ± 0.09 mm in controls (Supplementary Fig. S7). There were no significant differences in absolute width.

For juvenile herring, a broader suite of cardiac measurements was collected from the individuals used in *U*_*crit*_ assays (Table 2). As noted above, juveniles from the oil-exposed group were larger, with a higher condition factor (or K; K = 100 X (weight in g)/(length in cm)^3^) than controls (Table 2). Oil exposure altered cardiac morphology (Table 2), observed as significant increases in the ventricular lateral aspect ratio (controls 1.45 ± 0.02, oil-exposed 1.52 ± 0.02) and volume (controls 0.042 ± 0.003 mm^3^, oil-exposed 0.050 ± 0.002 mm^3^), as well as a reduction in the outflow tract angle (controls 21.9 ± 1.8°, oil-exposed 15.4 ± 1.4°). There was no significant change in the normalized lateral length or width, the ventral dimensions or aspect ratio, or the length of the bulbus arteriosus (Table 2). As indicated in Table 2, several anatomical measures in herring showed significant tank effects, both in the presence and absence of a significant effect of oil exposure. However, post-hoc statistical tests indicated that tank effects were unlikely to confound any of the significant oil exposure effects, because there was no single tank consistently associated with a tank effect. For example, although higher condition factor was associated previously with different ventricular shape in adult rainbow trout^23^, here the tank with the most different condition factor (C11) was not the same as the tanks with the most different aspect ratio (O8) or ventricular volume (C6).

**Figure 4:**
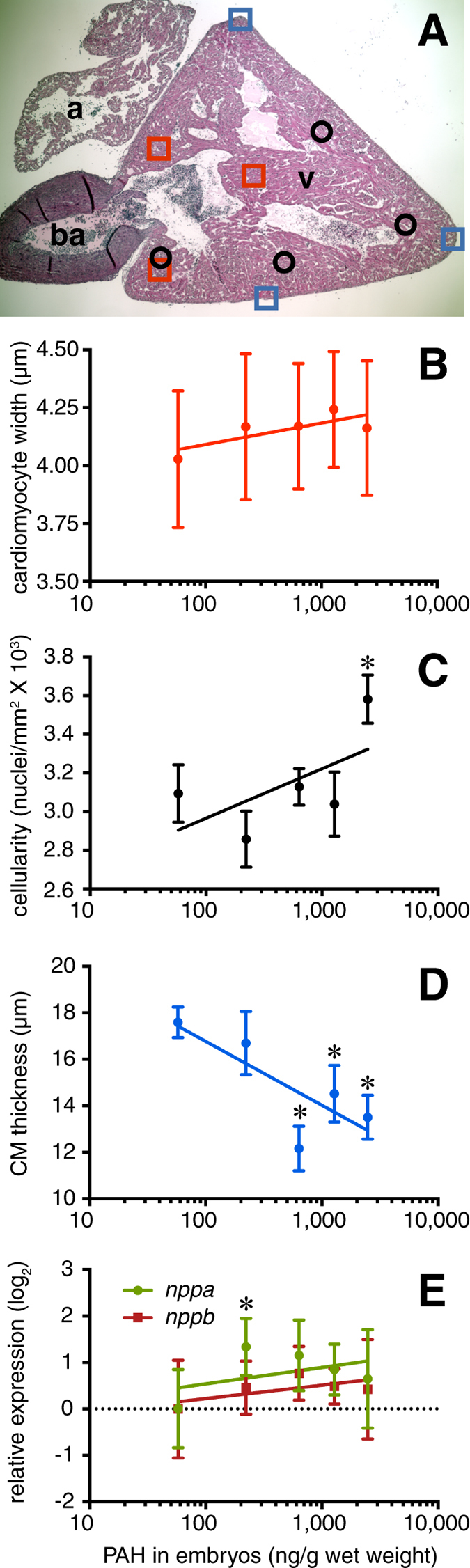
Distinct changes in spongy and compact myocardium in juvenile pink salmon following embryonic oil exposure. A subset of hearts from fish 8 months post-exposure for which dimensions were measured in Fig. 3 were sectioned and stained with hematoxylin/eosin. (**A**) Sections were selected that showed the atrium (a) and ventricle (v), with a clear midline plane through the bulbus arteriosus (ba) and ventricular-bulbar valve. (**B**) Cardiomyocyte diameters, (**C**), numbers of cardiomyocyte nuclei and (**D**) thickness of the compact myocardium were measured in the indicated areas (**A**, red squares, black circles, and blue squares respectively). (**E**) Levels of mRNA for atrial (*nppa*) and B-type natriuretic peptide (*nppb*) in pink salmon hearts normalized to the reference gene *ef1α*. All data are mean ± s.e.m. ANOVA showed significance for effect of oil exposure for compact myocardium thickness (*P* = 0.02) and density of nuclei (*P* < 0.0001). Asterisks indicate treatments that differed significantly from controls (Dunnett’s post-hoc test, α = 0.05).

Changes in ventricular shape could be indicative of cardiac hypertrophy. Extensively characterized in mammals^24^ and occurring through similar pathways in salmon^25^, cardiac hypertrophy is initially a compensatory response to cardiac stress or injury. At the cellular level, hypertrophy in mammals is indicated primarily by changes in cardiomyocytes that occur concentrically (increased cross-sectional area of individual cardiomyocytes), or eccentrically (lengthening of individual cardiomyocytes). However, fish hearts have the capacity to regenerate cardiomyocytes^26^, and hypertrophy in fish also involves hyperplasia^25^,^27^. We examined hearts dissected from pink salmon eight months post-exposure for histopathological evidence of hypertrophy. The fish ventricle consists of two anatomically distinct sections, an outer compact myocardium surrounding a spongy myocardium with numerous trabeculae projecting into the lumen, the latter making up the bulk of the ventricle. Individual cardiomyocyte lengths (eccentric hypertrophy) could not be measured in the spongy myocardium, but cardiomyocyte diameters were measured in cells randomly selected from three consistent areas (Fig. 4A). Analysis in fixed sections from 5–6 individuals for each treatment group showed a trend of increased cardiomyocyte width related to oil exposure with a mean (±s.e.m.) in controls of 4.03 ± 0.05  μm and ranging from 4.16 ± 0.05 to 4.24 ± 0.05  μm across oil-exposed groups (Fig. 4B). Although statistical significance was marginal (ANOVA *P* = 0.04 for effect of oil exposure, but slope of linear regression *P* = 0.1), this may be due in part to the relative insensitivity of the technique^24^. Oil exposure was associated significantly with hyperplasia in the spongy myocardium (ANOVA *P* < 0.0001), with an increase in the density of cardiomyocyte nuclei in the highest exposure group (123% of control, *P* = 0.0002; Fig. 4C). In contrast, the thickness of the compact myocardium was reduced significantly by oil exposure in all but the lowest treatment group (ANOVA *P* = 0.02; Fig. 4D), with control hearts averaging 17.6 ± 0.7  μm thick, and oil-exposed groups ranging from 12.2 ± 1.0 to 16.7 ± 1.4  μm. Other types of reactive changes were not obvious. We examined the mRNA expression levels of atrial and B-type natriuretic peptides (ANP/*nppa* and BNP/*nppb*) in salmon hearts, as these genes are strongly linked to cardiac hypertrophy in humans^28^. Although there was a trend of increasing *nppa* and *nppb* mRNA levels with ΣPAH in pink salmon (Fig. 4E), there was no statistically significant dose-response relationship, with only the lowest exposure group (tissue ΣPAH 222 ng/g) showing significantly higher expression of *nppa* at 2.5-times above control (*P* = 0.04, Dunnett’s method).

### Delayed cardiotoxicity relative to measured PAH levels in oiled habitats

We accessed the publicly available database for PAH concentrations measured in water samples collected in Prince William Sound during the 1989 herring spawning season. During the period of shoreline herring egg deposition (March 31–May 18, 1989), a total of 233 water samples were collected in or near the spill zone at a depth of 1 to 5 m (Supplementary Database S1). Of these, only two had ΣPAH values above 10  μg/L, while 108/233 (46%) had ΣPAH values between 1  μg/L and 10  μg/L. However, 98% of the surface water samples (229/233) had PAH concentrations that were at or above the ΣPAH 0.23  μg/L level that caused prolonged developmental cardiotoxicity among herring in the current study. Confined to the measured concentrations of three-ringed PAHs (sum of parent and alkyl fluorenes, dibenzothiophenes, and phenanthrenes), the effective exposure concentration observed here in herring was 0.15  μg/L, providing a more conservative comparison. Using this three-ringed PAH threshold, 108/233 water samples (46%) would be predicted to cause developmental cardiotoxicity as observed here.

## Discussion

Our findings indicate that embryonic exposure to very low, environmentally relevant levels of crude oil causes permanent structural and functional changes to the fish heart. Crude oil essentially acts as a potent teratogen that produces specific abnormalities in the compact myocardium and outflow tract following exposure during early heart development. These developmental defects initiated during organogenesis in turn led to reduced cardiorespiratory performance much later in juvenile fish. Hence, embryonic injury following crude oil exposure leads to irreversible impairment. Swimming performance in fish as measured by *U*_*crit*_ depends on oxygen uptake, delivery to and extraction by tissues^29^, and may be influenced by anaerobic metabolism^30^ or behavior^31^. While we did not directly measure cardiac output, non-cardiac influences on *U*_*crit*_ are extremely unlikely given (1) the known cardiotoxicity of crude oil-derived PAHs^16^, (2) the well characterized impacts of crude oil on cardiac morphology at concentrations lethal to fish early life stages^9^,^10^,^14^,^15^, (3) an embryonic exposure window that spanned a critical period in early heart development (e.g., looping) but ended before larval development of the gills and lateral line, and (4) measurable changes in cardiac anatomy and histology in the absence of overt neuromotor or behavioral alterations (e.g., unaffected cost of transport). Our cumulative and consistent findings across three very different species (zebrafish, herring and salmon) therefore support the conclusion that low-level embryonic crude oil exposures cause reduced cardiorespiratory fitness at later life stages.

In adult fish, an optimized ventricular shape confers a high cardiac output for prolonged swimming^23^,^29^,^32^,^33^ in species such as salmon and herring. While the relationship between ventricular dimensions and cardiac output has not been extensively characterized in juvenile fish, more active fish species generally have a thicker compact myocardium - e.g., salmon relative to demersal flatfish^33^. This extends also to comparisons within a single species; for example, thicker compact myocardium is associated with greater ventricular volume and higher swimming speed in juvenile Atlantic salmon (*Salmo salar*)^34^,^35^ and with greater migratory potential in adult sockeye salmon (*Oncorhynchus nerka*)^36^. The relationship of outflow tract (i.e., bulbus arteriosus) structure to cardiac output has not been defined, but both increased and decreased angles have been associated with abnormal ventricular shapes in zebrafish^37^ and salmonids^38^, respectively. Nevertheless, the impact of crude oil exposure on development of the compact myocardium alone is sufficient to explain reduced cardiorespiratory performance.

Although the molecular signaling events that underlie the changes in cardiac form observed here are likely different from pathological remodeling in human heart failure, our histological findings suggest that embryonic exposure triggered a compensatory hypertrophic response in spongy myocardium that is not matched by a similar response in the compact myocardium. Normal physiological remodeling in fish involves coordinated responses in both spongy and compact myocardium^25^. There is also a general positive correlation between overall fish size, heart size, and thickness of the compact myocardium^34^. The fact that embryonic oil exposure reduced growth and size of juvenile pink salmon here without a corresponding reduction in absolute heart size indicates a specific defect in development or growth of the compact myocardium. Fate mapping in zebrafish has shown that the compact myocardium of adult fish is clonally derived in the juvenile heart from a very small number of ventricular cardiomyocytes^39^. These precursor cells originate during embryonic development, within the oil exposure window for the present study^39^, and abnormal heart structure at later life stages may be a consequence of dysregulated clonal ontogeny. In our prior study with zebrafish, individuals assessed well into adulthood demonstrated rounder ventricles^11^, as opposed to the elongated ventricles observed here in juveniles. In developing zebrafish, the ventricle transitions from an elongated form in juveniles to a more rounded adult shape^37^. This suggests that a developmental defect in the compact myocardium causes an initial hypertrophic response that continues throughout life, ultimately leading to an abnormal, more extremely rounded ventricular form as individuals approach senescence.

Altered cardiac function is likely to influence juvenile health and survival both directly and indirectly. We were unable to directly measure respiration in juvenile pink salmon; however, embryonic oil exposure reduced juvenile growth in this and prior studies^18^, consistent with delayed effects on basal metabolic rate^40^. Size-dependent mortality is well established for juvenile marine fish^41^. Moreover, poor swimming performance (and by inference suboptimal cardiac function) is associated with reduced infectious disease resistance^42^. Thus, the delayed mortality previously reported from large-scale mark and recapture studies^17^,^18^ is a likely consequence of two forms of crude oil toxicity. The first encompasses direct effects on heart development, cardiac function, swimming performance, and associated consequences for prey capture and predator avoidance. The second is an indirect effect on basal metabolism, energy budget, growth, overall health and size-dependent predation.

Rates of larval and juvenile survival determine recruitment variability for most marine fish species. As such, they have a critical influence on population dynamics^43^, with small changes producing outsized impacts on recruitment^44^. Recent ecosystem modeling approaches that couple food webs and fish populations have shown via bioenergetics modeling that the metabolism and respiratory function of individual fish strongly influence population-scale dynamics, particularly for continuously swimming planktivorous clupeiforms like Pacific herring^45^,^46^. Our current findings demonstrate an interrelationship between crude oil cardiotoxicity, heart development, and the physiological parameters that determine larval-juvenile survival and, by extension, recruitment and population abundance.

Our findings shed new light on the ecosystem-scale crash of the Prince William Sound herring population several years after the Exxon Valdez spill. Previous analyses of the potential toxic impacts of the spill on herring spawn have considered only toxicity effects that occur above 1  μg/L^47^. We have shown that cardiac injury occurs in normal-appearing fish that survive even lower level PAH exposures, with a no-effect concentration yet to be established for herring. Moreover, studies of Pacific herring spawned in the wake of the 2007 Cosco Busan oil spill demonstrated that visible cardiotoxicity in live embryos (i.e., pericardial edema) was a more sensitive indicator of the presence of oil than visual surveys used to assess the extent of shoreline oiling both immediately after a spill and after cleanup efforts^48^. Accordingly, the impacts of the Exxon Valdez oil spill on populations of nearshore spawning fish are likely to have been considerably underestimated, in terms of both the geographic extent of affected habitats and the lingering toxicity of low levels of residual oil. It is also likely that the juvenile-to-adult recruitment from the 1989 herring year class was much lower than previously appreciated. More broadly, the exquisite sensitivity of the developing fish heart to PAHs also has implications for other pollution sources in marine ecosystems, including increasing maritime vessel traffic and expanding land-based urban runoff. These studies indicate the importance of understanding the long-term physiological consequences of chemical contaminant exposure. Addressing ecosystem-level changes that are delayed in time will necessitate integration across biological scales, from adverse impacts on individuals to population-level effects. This in turn will require novel coordination and collaboration between ecotoxicologists, physiologists, and population biologists.

## Methods

### Embryo production, oil exposure and post-exposure culture

Handling of adult fish, gamete isolation, fertilizations, and fish culture were all carried out according to the policies and guidelines of the U. S. Department of Commerce for aquaculture and experimental work with fish. Experimental design was approved by the Prince William Sound Regional Citizens’ Advisory Council Scientific Advisory Committee. Pink salmon eggs and milt were collected from nineteen females and fourteen males at the NOAA Auke Creek Hatchery, Alaska, on August 23, 2010. Eggs from each female were fertilized with milt pooled from three males, using standard hatchery procedures. Fertilized eggs were randomly mixed for each oil treatment level with roughly equal contribution from each female (mean 5,854 ± 89 eggs per incubator).

Ripe Pacific herring were captured by cast net on March 24, 2010 from a spawning aggregation in northern Puget Sound, WA (48° 6′ N–122° 31′ W) under permit from the Washington Department of Fish and Wildlife (Scientific Collection Permit 10–017). Gonads were dissected from 45 females (length 17.8 ± 0.4 cm, weight 58.4 ± 2.5 g) and 12 males (length 18.9 ± 0.3 cm, weight 61.3 ± 2.2  g). A total of 527 g ovaries (mean 11.7 ± 0.7  g each) were dissociated and the eggs pooled. Testes (104 g total, mean 8.7 ± 0.9  g each) were pooled and macerated in herring Ringer’s solution (206 mM NaCl, 7.2 mM KCl, 2.1 mM CaCl_2_, 3.1 mM MgCl_2_·6H2O, pH 7.6). Fertilization and controlled adherence onto nylon mesh was performed as described elsewhere^49^. Fertilization rates were 95–98% based on counts of cleavage stage embryos 24 hours after fertilization.

The overall experimental design for embryonic exposure and post-exposure growth is shown in Supplementary Fig. S1. Embryos of both species were exposed to effluent from oiled gravel columns prepared as previously described^7^,^8^,^50^. Five separate columns with graded amounts of oil (no oil, 0.5, 1, 2, and 3  g oil/kg gravel) were used for pink salmon exposures, while herring were exposed to effluent from a control column (no oil) or a single oil treatment (6 g/kg, previously weathered for 10 days^14^). Pink salmon embryo exposure began 1 h after fertilization and extended 50 days to the eyed stage. Embryos were hatched in a vertical stack incubator, and after yolk absorption emergent juveniles (about 3,300 per treatment) were transferred to 300-L tanks containing seawater. Juvenile fish were fed pelletized food (Bio-Oregon, Longview, WA) *ad libitum* beginning with transfer. Subsamples of approximately 50 fish per treatment were weighed and measured at 1–4 week intervals without replacement until 372 days post fertilization (dpf).

Fertilized Pacific herring eggs were adhered to nylon mesh sheets (n = 3 per treatment) and 1 h after fertilization suspended in 90-L aquaria receiving column effluent (~80 L reservoir) maintained at 9.1 ± 0.1 °C. At 8 dpf, embryos were subsampled for live morphological analysis, RNA extraction, and PAH tissue analysis, then suspended in randomized 600-L tanks with filtered seawater flowing at 1 L/min for hatching. Hatching commenced at 14 dpf and was complete by 20 dpf, with 4.7 ± 0.9% and 2.7 ± 0.4% of eyed embryos unhatched in control and oiled tanks, respectively. Larvae were fed rotifers enriched with phytoplankton (*Nannochloropsis* and *Pavlova* algae paste, live *Tetraselmis* and *Nichia*; Brine Shrimp Direct, Ogden, UT) on days 1–20, with newly hatched *Artemia* added at day 5. After day 20 the diet was switched to frozen copepods (Cyclop-eeze, Argent Laboratories, Redmond, WA) and krill.

### Analytical chemistry

Hydrocarbons were extracted from water and tissue with dichloromethane, dried, fractionated, purified, and processed by gas chromatography-flame ionization detection (GC-FID) and gas chromatography-mass spectroscopy (GC-MS) as detailed elsewhere^11^,^51^. Briefly, water was extracted on days 0, 1, 2, 10, 21, 32, and 50 for pink salmon and days 0 and 8 for herring. Extraction volumes ranged from 1 to 3.8 L for pink salmon depending on expected aqueous PAH concentrations and were 150  mL for herring. Embryo samples for salmon were 6.9 ± 0.3 g wet weight, taken at exposure days 0, 21 (predicted peak uptake), and 50, and for herring were 1.5 ± 0.3  g wet weight taken at day 8. The data were acquired in selected ion monitoring (SIM) mode and PAH concentrations determined by the internal standard method^51^. Experimentally determined method detection limits were about 1 ng/L (pink salmon) to 17  ng/L (herring) in water and about 0.2 ng/g (pink salmon) to 0.8  ng/g (herring) in tissue. PAH concentrations in Prince William Sound water samples were obtained from the Exxon Valdez Trustee Hydrocarbon Database (URL). Using Microsoft Excel, values for individual analytes were summed for total ΣPAH and total tricyclic compounds (parent and alkyl fluorenes, dibenzothiophenes, and phenanthrenes), and filtered to rank samples by date and descending summed concentration.

### Swimming performance and respirometry

Swimming performance was measured with a 1.5-L swim tunnel respirometer (model SW10040, Loligo Systems, Tjele, Denmark) in seawater at 8–9 °C. Pink salmon assays were limited to three treatment levels (control, ΣPAH 15  μg/L, and ΣPAH 45  μg/L) due to the tradeoff between assay duration and ongoing growth. For both species, fish were captured from treatment groups randomly with the swim tunnel operator blind to treatment. Single fish were acclimated for 30 minutes at a flow rate of 5 to 10  cm/sec; rates were then increased by 5 cm/sec every 5 minutes for salmon and every 10 minutes for herring. Failure was defined as impingement on the downstream baffle for >1 s. Each impinged fish was given a chance to resume swimming at a lower water velocity, and then quickly returned to the failure speed. The first or second failure time was recorded depending on whether the fish resumed swimming, and rounded to the nearest minute. *U*_*crit*_ was calculated with the formula V_f_ + (T/*t*)*dV*, where V_f_ is the highest velocity maintained for a full interval, T is the time spent at the final velocity, *t* is the interval between velocity increments, and *dV* the incremental change in velocity. The maximum tunnel flow rate was 50 cm/sec, and fish that reached or exceeded this velocity were allowed to swim without time restriction until failure (8% of juvenile pink salmon). Oxygen consumption was measured for herring by recording the dissolved oxygen level (mg/L) at the end of acclimation and at each velocity increment up to failure. Linear regressions between time and dissolved oxygen concentration were calculated with Microsoft Excel, and slopes with *r*^*2*^ ≥ 0.9 were used to calculate oxygen consumption based on the respirometer volume (1.5 L) and fish mass. Cost of transport^52^ was calculated using a fish oxycaliforic equivalent of 14.1 J/mg O_2_ multiplied by maximum metabolic rate and divided by *U*_*crit*_.

### Cardiac anatomy and histology

For pink salmon, heart shape was assessed in animals randomly selected from each treatment group at the beginning and end of the swim assays (July 6–8 and September 8–9 2011, respectively). For Pacific herring, heart shape was assessed in individuals with corresponding *U*_*crit*_ measures. Following sacrifice by MS-222 overdose, hearts of herring and smaller salmon were excised at the ventral aorta and sinus venosus, positioned in a petri dish flooded with either herring Ringer’s solution or phosphate-buffered saline, and imaged on a Nikon SMZ800 or Wild M5A stereomicroscope fitted with FireI-400 (Unibrain, San Ramona, CA) or QImaging Micropublisher 5.0 RTV (Surrey, BC, Canada) digital camera, respectively. For larger salmon (September), MS-222 overdosed fish were dissected to expose the lateral aspect of the ventricle, and hearts were imaged *in situ* with a digital camera using a macro lens setting. Images of dissected hearts were calibrated with a stage micrometer, allowing absolute measurements of cardiac dimensions (ventricular length and width, bulbus arteriosus length). Relative measurements only (aspect ratio) were obtained from hearts imaged *in situ*. All anatomical measurements were made with ImageJ (rsbweb.nih.gov/ij/).

Formalin-fixed pink salmon hearts were paraffin-embedded and sectioned as described elsewhere^11^. Hearts were embedded in the same dorsal-ventral orientation and cellular measurements taken in sections from the same mid-line plane containing the ventricle lumen, bulbus arteriosus and ventriculobulbar valve. Sections were imaged on a Nikon Eclipse 50i or Nikon E600 compound microscope and measured using ImageJ. Cardiomyocyte diameters were obtained from 10 cells each in three areas of the ventricular wall in three sections of each heart (90 cells total from each heart). Thickness of compact myocardium was measured in three areas of the ventricle for three sections from each heart. Cardiomyocyte nuclei were counted in four areas of spongy myocardium in each of three sections; total nuclei were counted in images that covered 225  μm^2^ by 300  μm^2^.

### cDNA cloning and QPCR Analysis

Clones for atrial and B-type natriuretic peptide mRNAs (*nppa* and *nppb*, respectively) were isolated from juvenile pink salmon hearts, while herring *cyp1a* (encoding cytochrome P4501A) was isolated from ANSCO-exposed embryos^14^, using reverse transcription polymerase chain reactions (RT-PCR) and rapid amplification of cDNA ends (RACE) (primers^53^,^54^ provided in Supplementary Table S2). GenBank accession numbers for gene sequences are provided in Supplementary Table S3. Gene-specific RT-QPCR (QPCR) primers (Supplementary Table S3) were designed using Primer3 (http://www-genome.wi.mit.edu/genome_software/other/primer2.html), and synthesized by Sigma-Aldrich (St. Louis, MO). Total RNA was extracted from pink salmon hearts and herring embryos in TRIzol and QPCR was performed on an ABI 7700 Sequence Detector (Applied Biosystems) using Power SYBR Green Master Mix (salmon *nppa* and *nppb*,) or SYBR Select Master Mix (herring *cyp1a*). Target genes were normalized to a reference gene, *ef1a*^55^, after confirming equal efficiencies of target and reference gene assays, and stable expression of *ef1a* across control and treatment samples.

### Statistical analysis

Analyses of pink salmon growth and swimming performance data were conducted using SAS (SAS Institute, Cary, NC) and Minitab (Minitab, Inc., State College, PA), and involved analysis of variance (ANOVA; one- or two-factor) to determine statistical significance, regression for relationships between dependent and independent variables (significance determined by Pearson’s correlation coefficient and F-tests) and analysis of covariance (ANCOVA) to evaluate response to treatment. Dose-response data for pink salmon hearts (morphology and histology) were analyzed using linear and non-linear regressions in Prism 6.0b for Macintosh (Software McKiev, Boston, MA). Non-linear models were compared to linear models statistically, and most data fit straight-line models. ANOVA and post-hoc analyses (Dunnett’s method) were applied using JMP 8.0.1 for Macintosh (SAS Institute, Cary, NC), with simple one-way ANOVA for single measurements from individuals (e.g., aspect ratio), and nested ANOVA for measurements from multiple histological sections (individual fish nested within treatment). Data for herring measures were analyzed by one-way ANOVA with replicate (tank) nested within treatment (exposure), with Student’s t and Tukey Kramer Honestly Significant Differences tests for post-hoc means comparisons for treatment effect and tank effect, respectively.

## Additional Information

**How to cite this article**: Incardona, J. P. *et al*. Very low embryonic crude oil exposures cause lasting cardiac defects in salmon and herring. *Sci. Rep*. **5**, 13499; doi: 10.1038/srep13499 (2015).

## Supplementary Material

Supplementary Information

Supplementary Database S1

## Figures and Tables

**Table 1:** PAH concentrations in exposure water and embryonic tissues of pink salmon and herring.

Species	Treatment	ΣPAH water (μg/L)	ΣPAH in embryos(ng/g wet weight)	ΣPAH in embryos(ng/g lipid)
Pink salmon	clean gravel	0.2	26	240
	0.5 g/kg oiled gravel	9.8	222	2,066
	1 g/kg oiled gravel	15.4	634	5,895
	2 g/kg oiled gravel	30.0	1,279	11,900
	3 g/kg oiled gravel	45.4	2,474	23,012
Pacific herring	clean gravel	0.039 ± 0.003	9.3 ± 3.7	582 ± 178
	oiled gravel	0.230 ± 0.010	28.6 ± 10.2	1,787 ± 256

**Table 2:** Anatomical measures in juvenile Pacific herring.

measure	control	oil exposed	*P* oil effect^b^	*P* tank effect^c^
Fork length (cm)	5.3 ± 0.1	5.6 ± 0.1	0.0005	0.0007 (C6)
Mass (g)	1.26 ± 0.07	1.65 ± 0.10	0.0003	0.0004 (O2)
Condition factor	0.82 ± 0.01	0.87 ± 0.01	0.01	0.02 (C11)
Ventricle length—lateral^a^	69.5 ± 1.0	69.8 ± 1.2	0.2	0.001 (O8)
Ventricle width—lateral^a^	51.3 ± 0.9	49.8 ± 0.7	0.7	0.3
Aspect ratio—lateral	1.45 ± 0.02	1.52 ± 0.02	0.04	0.03 (O8)
Ventricle length—ventral^a^	64.0 ± 1.0	63.6 ± 1.5	0.6	0.004 (O8)
Ventricle width—ventral^a^	63.5 ± 0.9	64.3 ± 1.0	0.07	0.01 (O8)
Aspect ratio—ventral	1.14 ± 0.01	1.13 ± 0.01	0.2	0.003 (C11)
Ventricle volume (mm^3^)	0.042 ± 0.003	0.050 ± 0.002	0.007	0.002 (C6)
BA length (relative to ventricle)	0.45 ± 0.01	0.47 ± 0.01	0.6	0.5
OFT angle (degrees)	21.9 ± 1.8	15.4 ± 1.4	0.004	0.5

^a^Normalized to fork length.

^b^Data analyzed by one-way ANOVA with tank nested under treatment (oil exposure).

^c^Parentheses indicate identity of statistically different tanks, e.g., C6 = control tank 6, O2 = oiled tank 2.
