# Supplementary material for: Very low embryonic crude oil exposures cause lasting cardiac defects in salmon and herring
Source: Sci Rep. 2015 Sep 8;5:13499. doi: 10.1038/srep13499 (PMC4561892; doi:10.1038/srep13499)
Supplement: Supplementary Information [file srep13499-s1.pdf]

Very low embryonic crude oil exposures cause lasting cardiac defects in salmon and herring

John P. Incardona, Mark G. Carls, Larry Holland, Tiffany L. Linbo, David H. Baldwin, Mark S. Myers, Karen A. Peck, Mark Tagal, Stanley D. Rice, and Nathaniel L. Scholz

Supplementary Information

Figures S1-S7

Tables S1-S3

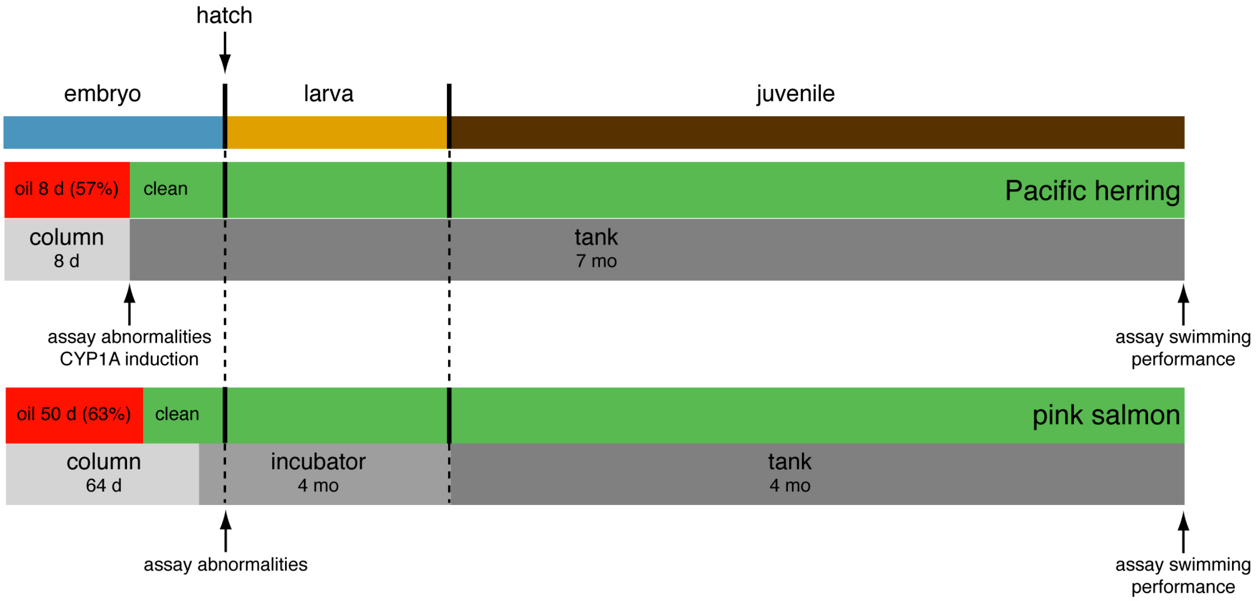

Figure S1. Experimental design for embryonic exposure and post-exposure growth. The top bar demarcates the embryonic (blue), larval (amber) and juvenile (brown) life stages relative to exposure and growth. Relative length of each stage is arbitrary and does not represent actual time for a given stage. The middle and lower bars indicate the relative exposure and rearing periods for herring (middle) and pink salmon (lower), and points where measurements were made (arrows). Red indicates the proportion (%) of embryonic development during which each species was exposed to oiled-gravel effluent, with absolute exposure time given for each species in days (d); green indicates the period spent in clean water. Gray bars provide the time spent in different culture environments. Exposure and rearing paradigms were tailored to the different life history strategies of each species, e.g., herring as nearshore demersal spawners that produce pelagic larvae and salmon as anadromous spawners that deposit eggs in streambed nests, within which embryos complete larval development. Herring embryos were exposed in gravel effluent reservoirs, then transferred to rearing tanks to complete embryonic development, hatch, complete larval development and juvenile growth. Salmon embryos were maintained in gravel effluent reservoirs with switching of oiled effluent to clean effluent, followed by transfer to a vertical stack incubator for hatching and completion of larval development (yolk absorption), followed by transfer to rearing tanks for juvenile growth.

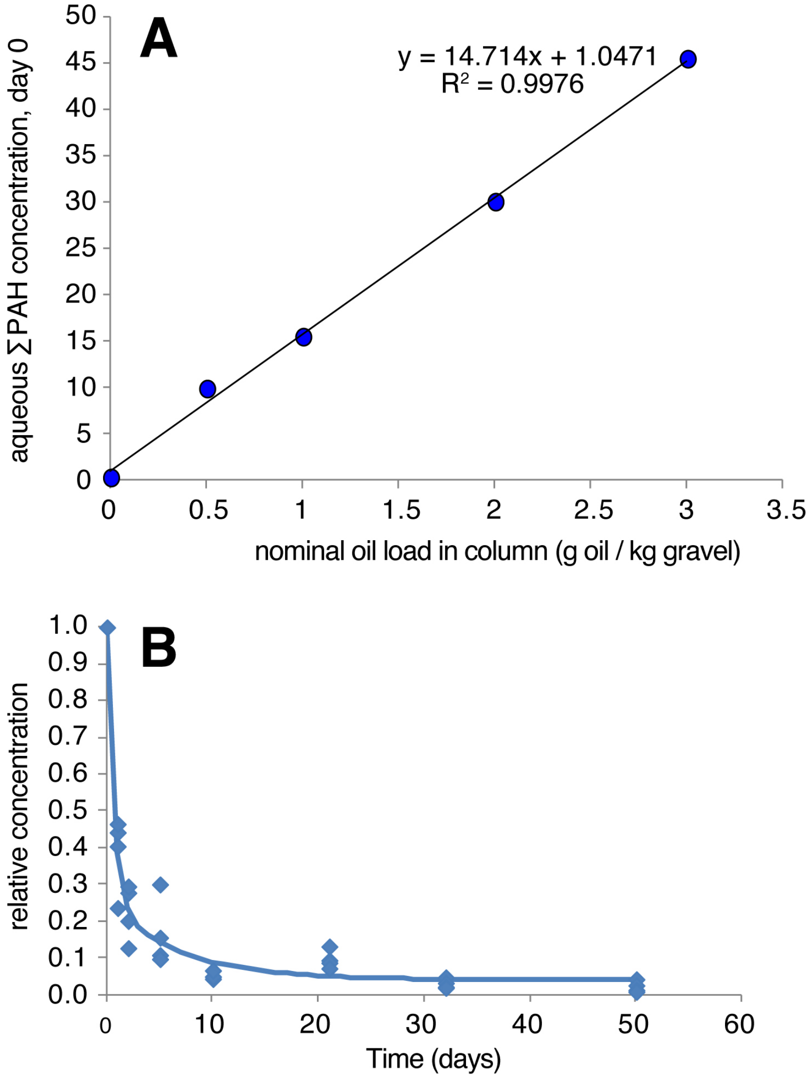

Figure S2. Total aqueous PAH concentrations in column effluents during pink salmon exposure. (A) Total sum (∑) PAHs in column effluent (µg/L) at the beginning of exposure (day 0) as a function of oil mass applied to gravel. Linear regression results are provided above the line. (B) Relationship between ∑PAH aqueous concentration and time. All data from the five exposure levels were combined in this analysis, each normalized to initial aqueous concentration.

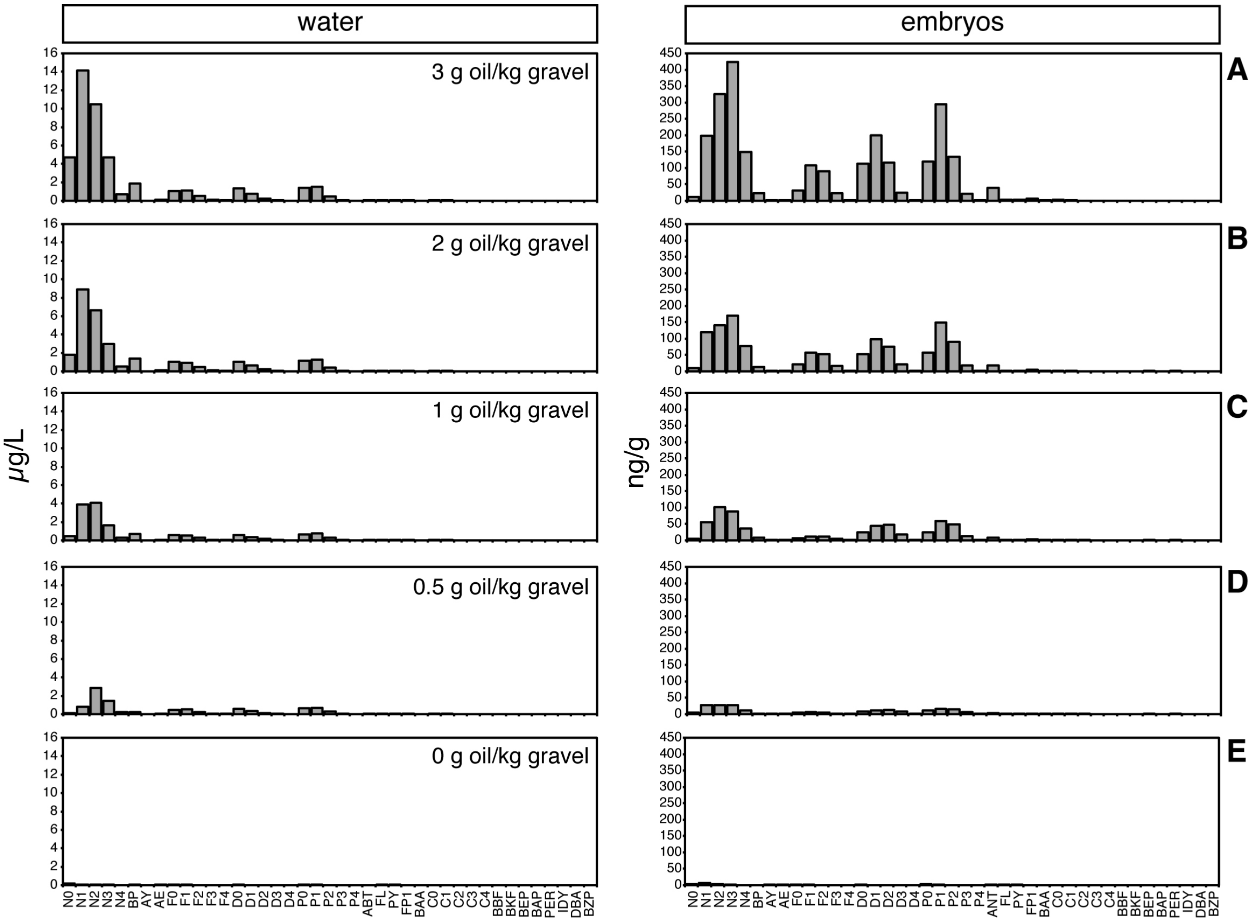

Figure S3. Individual PAH concentrations in column effluent and pink salmon embryos. PAH concentrations are shown for column effluents at the start of exposure and in embryos at exposure day 21 at all treatment levels. Left panels show aqueous PAHs measured in single water samples, right columns show PAHs measured in embryos from single pooled samples (each ≥7 g tissue wet weight). (A) 3 g/kg oil load, (B) 2 g/kg oil load, (C) 1 g/kg oil load, (D) 0.5 g/kg oil load. (E) control clean gravel. N, naphthalenes; BP, biphenyl; AY, acenaphthylene; AE, acenaphthene; F, fluorene; D, dibenzothiophene; P, phenanthrene; ANT, anthracene; FL, fluoranthene; PY, pyrene; FP, fluoranthenes/pyrenes; BAA, benz[*a*]anthracene; C, chrysene; BBF, benzo[*b*]fluoranthene; BKF, benzo[*j*]fluoranthene/benzo[*k*]fluoranthene; BEP, benzo[e]pyrene; BAP, benzo[a]pyrene; PER, perylene; IDY, indeno[1,2,3-*cd*]pyrene; DBA, dibenz[*a*,*h*]anthracene/dibenz[*a*,*c*]anthracene; BZP, benzo[*ghi*]perylene. Parent compound is indicated by a 0 (e.g., N0), while numbers of additional carbons (e.g. methyl groups) for alkylated homologs are indicated as N1, N2, etc.

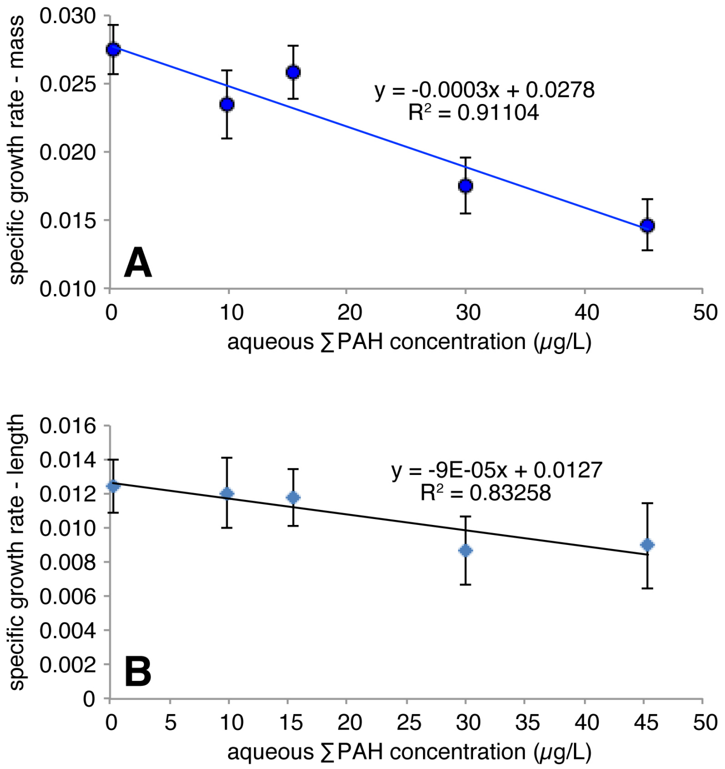

Figure S4. Reduced growth rates in juvenile pink salmon following embryonic oil exposure. Specific growth rates as a function of embryonic aqueous ∑PAH concentration for mass (A) and length (B) were determined during growth up to the point of swim trials. Values are mean ± s.e.m. for ~ 250 fish at each dosing level, derived from weight and length measures on a minimum of 10 and up to 55 fish at each time point.

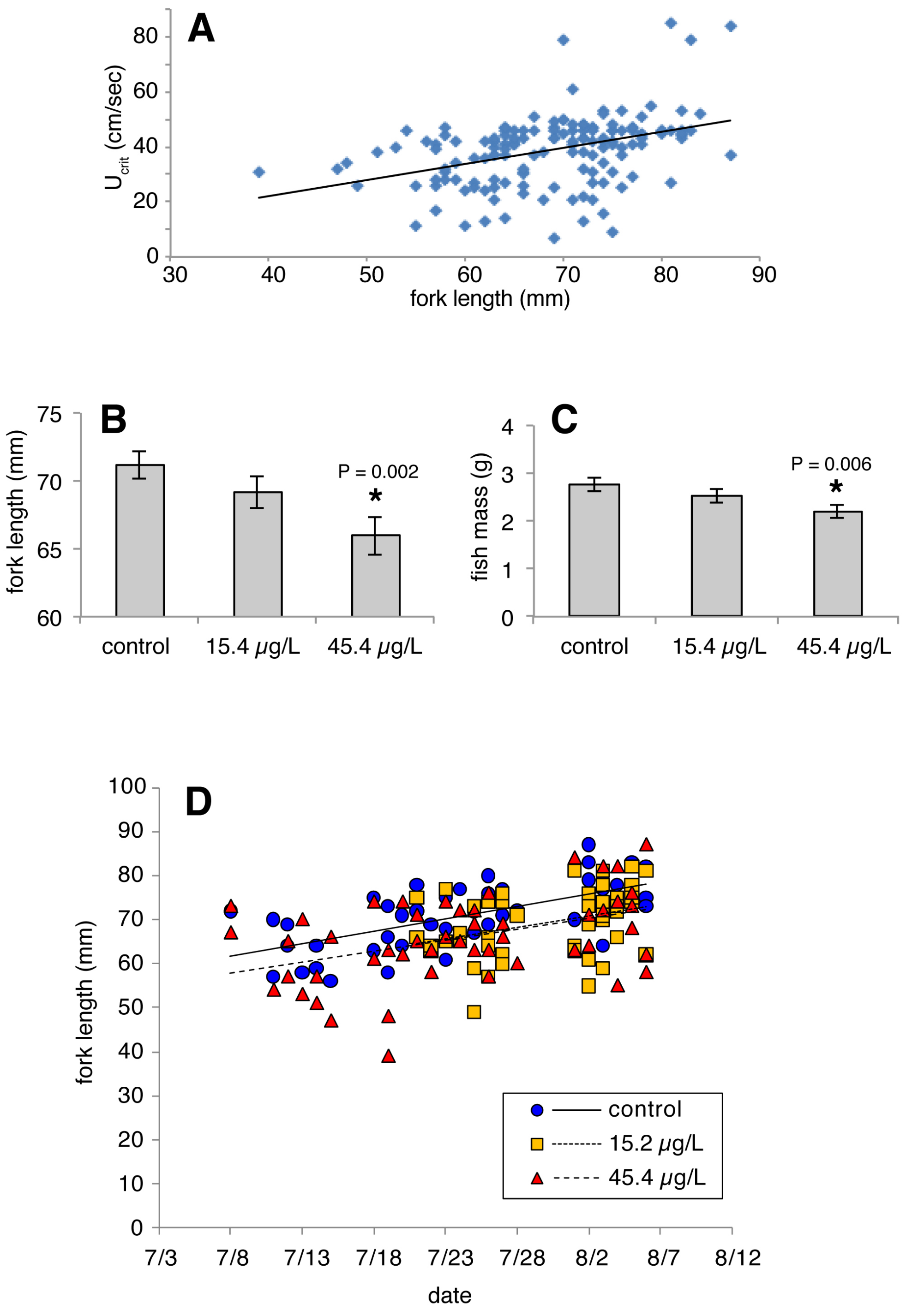

Figure S5. Fish size in swimming performance assays. (A) Relationship of absolute U_{crit} (cm/sec) to fish length for all fish assayed. Data represent individual U_{crit} measure and fork length of all fish from each of the three treatment groups assayed. (B) Fork length and (C) weight of individual assayed for each of three treatment groups. Data are mean ± s.e.m. (control, $N = 45$; 15 µg/L, $N = 52$; 45 µg/L, $N = 52$). Asterisks indicate statistically different groups determined by one-way ANOVA and post-hoc means comparison. (D) Length of fish as a function of swim trial date (July 3 through August 12).

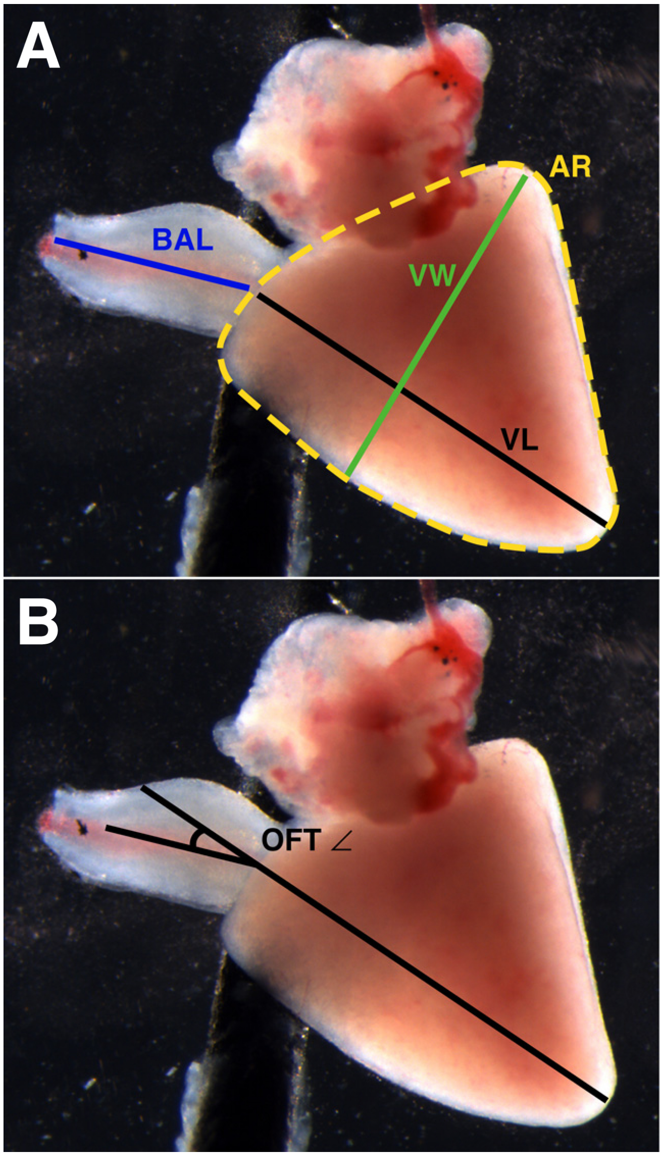

Figure S6. Locations and orientation for measure of cardiac dimensions. Hearts dissected from both pink salmon and Pacific herring were oriented and imaged as shown for a representative pink salmon heart (lateral view with anterior to the left). (A) Ventricular lengths were measured from the center of the bulbus arteriosus to the apex (black line, *VL*), while ventricular widths were measured from the dorsal apex perpendicular to the length line (green line, *VW*), Aspect ratio was measured by tracing the perimeter of the ventricle with the freehand line tool in ImageJ (yellow dashed line, *AR*); the application automatically determines the widest perpendicular axes to produce the calculated ratio. Length of the bulbus arteriosus was measured from the midline of where it joins the ventricle to the midline of the distal lumen at the dissection point (blue line, *BAL*). (B) The outflow tract was measured by drawing a line with one segment running from the apex to the midline of the ventricular – bulbar junction, and a segment running along the midline of the bulbus (*OFT* ∡).

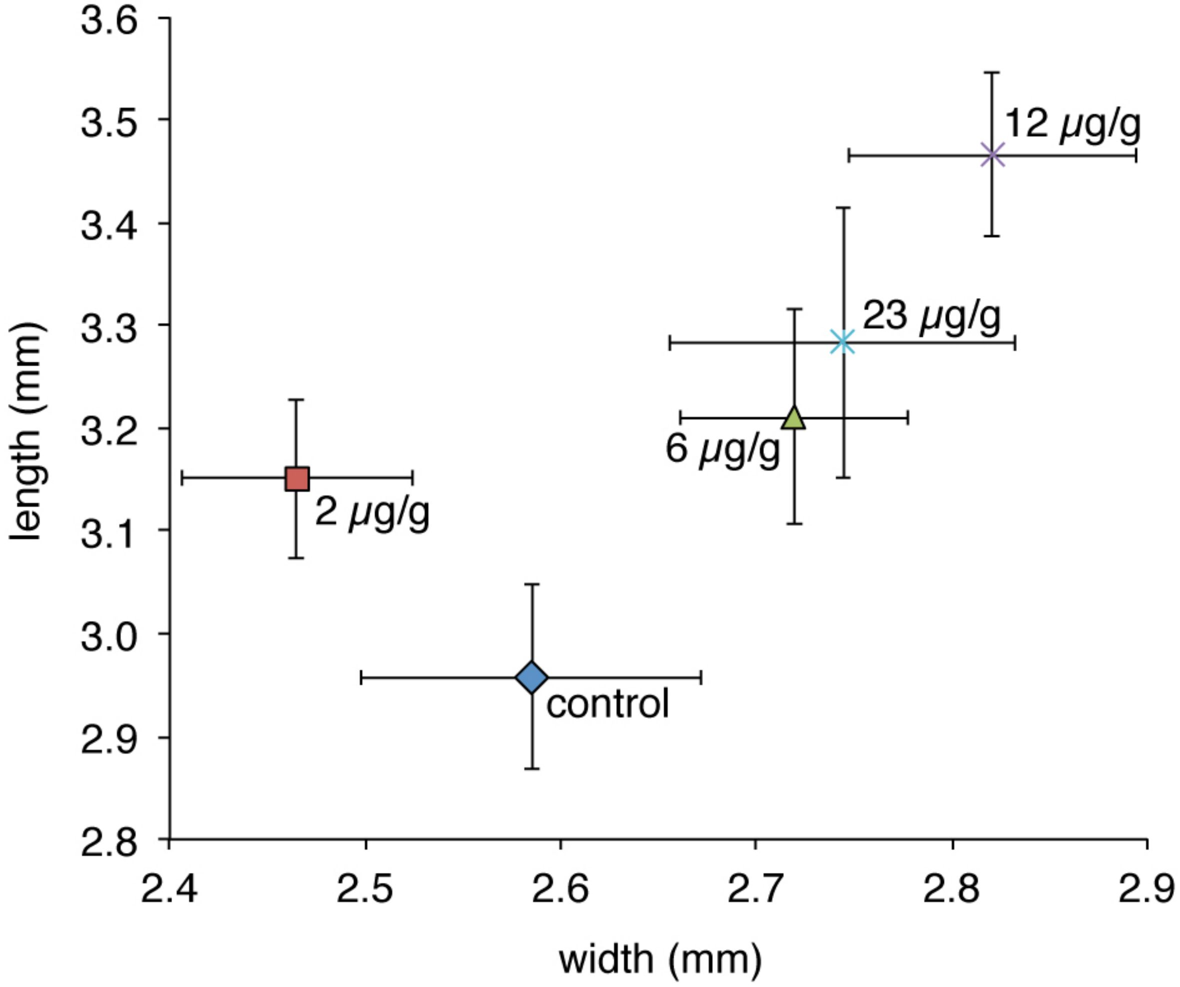

Figure S7. Absolute dimensions of pink salmon ventricles. Length and width (mm, mean ± s.e.m.) are plotted for all exposure groups at the 8-month sampling point, with tissue ∑PAH dose indicated by each data point. $N = 12$ for all oil exposed groups, 13 for control.

Table S1: Morphological signs of toxicity in exposed embryos

Species	∑PAH water (µg/L)	N	Pericardial edema (%)	Yolk sac edema (%)	Hemorrhage (%)
Pink salmon	0.2	96	0	1	0
	9.8	86	0	22	3
	15.4	120	9	38	7
	30.0	89	2	24	3
	45.4	88	11	33	9
Pacific herring	0.039 ± 0.003	68	1.2 ± 1.2	0	NA
	0.230 ± 0.010	81	11.8 ± 3.2	0	NA

Table S2. Genes and primers used in cDNA cloning, 5' to 3'

Gene		Primer
pink salmon		
nppa –	5' RACE[a]	GACTGCAGCCTAAACCGCTCGAGGTC
	3' RACE[a]	AGGACTGCCGTCTCATGGGGACTTCTG
nppb	5' RACE[b]	GTATTTGCCAACYGTGKTGCATCC
	3' RACE[b]	CGTATCCTGTCTACAAYGGGYTACTGAC
Pacific herring		
cyp1a	internal[c]	F:ATHGAYCAYTGYGARGAYMG
	internal[c]	R:TCYTTNGGDATRAARTANCC
	5' RACE[d]	TGAAGGCTTCCAAAAGAGGGAGGCTGG

[a]designed based on alignment of available teleost *nppa* sequences

[b]designed based on alignment of available teleost *nppb* sequences

[c]ref. 41, F = forward primer, R = reverse primer

[d]gene-specific primer

Table S3. Genes and primers used for RT-qPCR, 5' to 3'

Gene	Primer Pair	GenBank accession number or reference
pink salmon		
nppa – 5'	F: ACCAGGAGTAAAGCTGTGTCTG	KF271796
	R: GACTAGCTACGTCTTTTAGGACTGC	
nppa – 3'	F: ATCATAGAGGCACATTGACTGG	KF271796
	R: TGTGTGTCACTACACCCTCTCTC	
nppb	F: AATGAGCTCTCTTGGATGCAC	KF271797
	R: AGTAATGACGTTGCTGTTCCAG	
*ef1*α	F: CCCCTGGACACAGAGATTTCATC	ref. 44
	R: AGAGTCACACCGTTGGCGTTAC	
Pacific herring		
cyp1a	F: AGGAGCACATCAGCAAGGAG	KF271793
	R: ACCACCTGTCCGAACTCATC	
*ef1*a	F: CTGGTATGGTTGTGACCTTCG	DQ334851.1
	R: ACGGATATCCTTGACTGACACG	

F = forward primer; R = reverse primer
